# Evaluation of the immunization effectiveness of bOPV booster immunization and IPV revaccination

**DOI:** 10.1038/s41541-023-00642-w

**Published:** 2023-03-18

**Authors:** Zhao Yu-ping, Li Jing, Huang Teng, Ying Zhi-fang, Zhao Ting, Che Yan-chun, Zhao Zhi-mei, Fu Yu-ting, Tao Jun-hui, Yang Qing-hai, Wei Ding-kai, Li Guo-liang, Yang Xiao-lei, Yi Li, Chen Hong-bo, Wang Jian-feng, Jiang Rui-ju, Yu Lei, Cai Wei, Yang Wei, Xie Ming-xue, Yin Qiong-zhou, Pu Jing, Shi Li, Hong Chao, Deng Yan, Cai Lu-kui, Zhou Jian, Wen Yu, Li Hong-sen, Huang Wei, Mo Zhao-jun, Li Chang-gui, Li Qi-han, Yang Jing-si

**Affiliations:** 1 Institute of Medical Biology, Chinese Academy of Medical Science & Peking Union Medical College, Kunming, China; 2 National Local Joint Engineering Research Center for Biological Products of Viral Infectious Diseases, Kunming, China; 3 Kunming Science and Technology Innovation Centre for Research, Development and Industrialization of New Outbreaks and Emerging Highly Pathogenic Pathogens Vaccines, Kunming, China; 4 GuangXi Province Center for Disease Prevention and Control, Nanning, China; 5 National Institutes for Food and Drug Control, Beijing, China; 6 Liujiang District Center for Disease Prevention and Control, Liuzhou, China; 7 Liucheng County Center for Disease Prevention and Control, Liuzhou, China; 8 Rong’an County Center for Disease Prevention and Control, Liuzhou, China

**Keywords:** Clinical trials, Immunology

## Abstract

To provide a basis for further optimization of the polio sequential immunization schedule, this study evaluated the effectiveness of booster immunization with one dose of bivalent oral poliovirus vaccine (bOPV) at 48 months of age after different primary polio immunization schedules. At 48 months of age, one dose of bOPV was administered, and their poliovirus types 1–3 (PV1, PV2, and PV3, respectively)-specific neutralizing antibody levels were determined. Participants found to be negative for any type of PV-specific neutralizing antibody at 24, 36, or 48 months of age were re-vaccinated with inactivated polio vaccine (IPV). The 439 subjects who received a bOPV booster immunization at the age of 48 months had lower PV2-specific antibody levels compared with those who received IPV. One dose of IPV during basic polio immunization induced the lowest PV2-specific antibody levels. On the basis of our findings, to ensure that no less than 70% of the vaccinated have protection efficiency, we recommend the following: if basic immunization was conducted with 1IPV + 2bOPV (especially Sabin strain-based IPV), a booster immunization with IPV is recommended at 36 months of age, whereas if basic immunization was conducted with 2IPV + 1bOPV, a booster immunization with IPV is recommended at 48 months of age. A sequential immunization schedule of 2IPV + 1bOPV + 1IPV can not only maintain high levels of antibody against PV1 and PV3 but also increases immunity to PV2 and induces early intestinal mucosal immunity, with relatively good safety. Thus, this may be the best sequential immunization schedule for polio in countries or regions at high risk for polio.

## Introduction

In September 2015, the Global Polio Eradication Certification Committee announced the eradication of wild poliovirus type 2 (WPV2), prompting the World Health Organization (WHO) Strategic Advisory Committee to recommend a global discontinuation of using the type 2 polio vaccine component of oral poliovirus vaccine (OPV). In May 2016, 155 countries around the world, including China, simultaneously implemented a change in polio immunization strategies. They ceased the use of trivalent OPV (tOPV) in their sequential immunization schedule and began using only bivalent OPV (bOPV) against poliovirus types 1 and 3 (PV1 and PV3) to prevent and control polio. Importantly, they also prioritized including at least one dose of inactivated polio vaccine (IPV) in their sequential immunization schedule to, as much as possible, avoid vaccine-associated paralytic poliomyelitis and circulating vaccine-derived poliovirus (cVDPV) caused by poliovirus types 2 (PV2) in the live polio vaccine while maintaining immunity to PV2, thus minimizing the potential risk of PV2 outbreaks^1–5^. Several clinical studies^6–9^ have shown that the revised sequential polio immunization program is highly immunogenic, yielding high seroconversion rates and the induction of high neutralizing antibody titers against PV1 and PV3, and that increasing the number of IPV doses can effectively improve the PV2 immunogenicity. Notably, the basic immunization program with one dose of IPV and two doses of bOPV (1IPV + 2bOPV) induces relatively low levels of immunity to PV2. Currently, outbreaks of VDPV continue to occur in several countries and regions, with more cases of poliomyelitis being caused by VDPV than by WPV globally, and the incidence of VDPV, among which VDPV2 is most serious, is increasing^10–15^. Therefore, it is necessary to explore the immunogenicity and safety of different polio immunization programs, including the immunization time and effect of booster immunization, to provide scientific support for the improvement of polio sequential immunization schedules and to help eliminate polio or maintain polio-free status.

From September 2015 to December 2016, a phase III clinical study on the immunogenicity and safety of sequential immunization with PV1 and PV3 bOPV (human diploid cells) combined with IPV in 2-month-old infants was carried out in Liujiang District, Liucheng County, and Rong’an County in Liuzhou City, Guangxi Zhuang Autonomous Region, China^6^. The present study was conducted on the participants of this prior study to further evaluate the immune effect of bOPV booster immunization. The analysis conducted in this paper aimed to evaluate the effect of bOPV booster immunization to explore the rationality of booster immunization with bOPV at 48 months of age following different sequential polio immunization programs, especially those using Sabin strain-based IPV (sIPV), and to provide a basis for continuous improvement of the polio sequential immunization program.

## Results

### Subject participation and baseline demographic characteristics

**Fig. 1:**
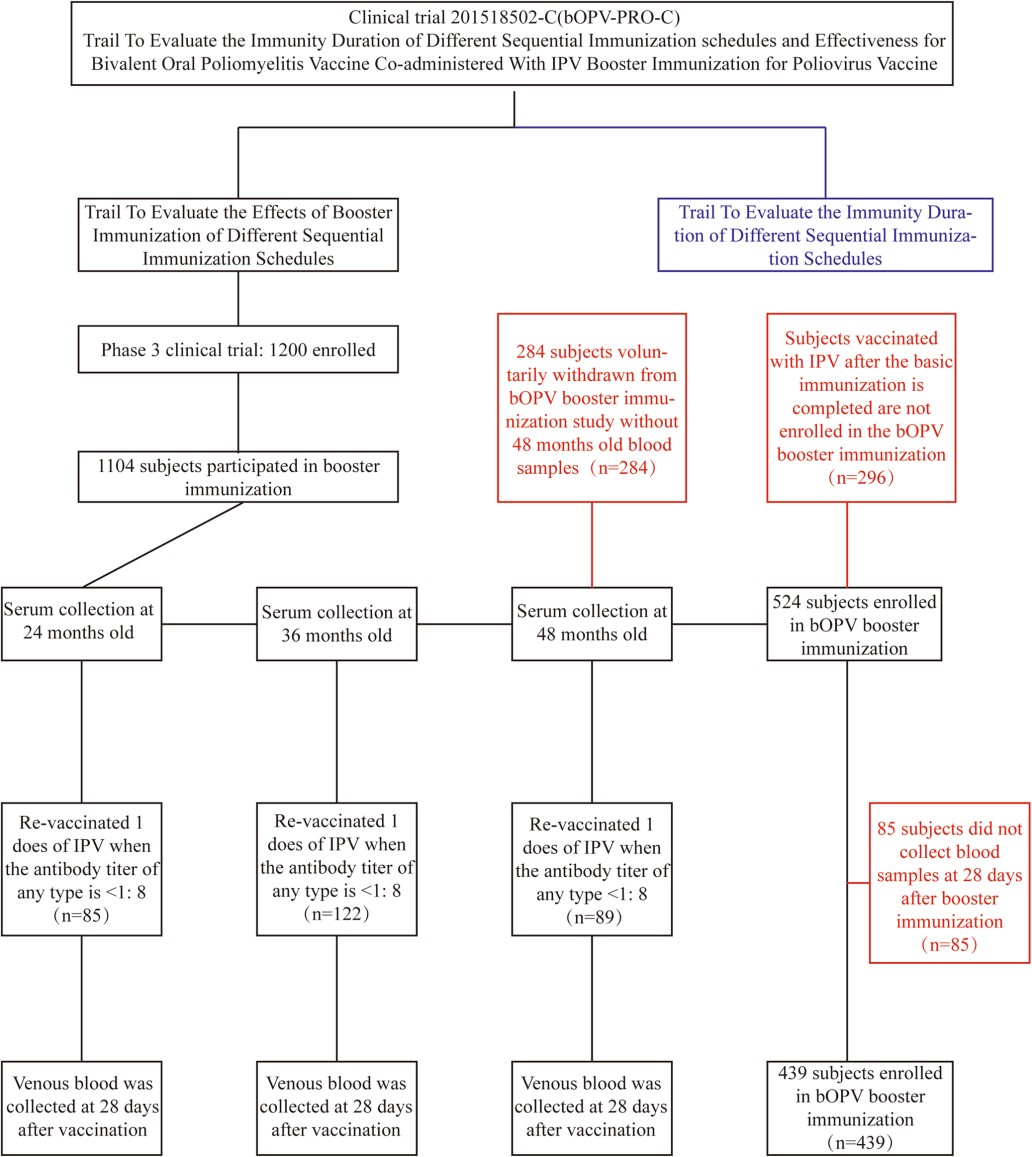
Flow chart of the study protocol for this polio booster immunization clinical trial. IPV inactivated poliovirus vaccine, bOPV bivalent oral poliovirus vaccine. Blue box: immunopersistence study, which is not within the scope of the present analysis; red boxes: descriptions of the situations of those who did not participate in this booster immunization clinical trial.

This analysis was conducted on data collected from a polio booster immunization clinical trial conducted in the Guangxi Zhuang Autonomous Region, China. Of the 1,200 volunteers who participated in the original phase III clinical trial: 96 withdrew (for various reasons, such as moving away), 85 were re-vaccinated with IPV at 24 months of age; 122 were re-vaccinated with IPV at 36 months of age; 89 were re-vaccinated with IPV at 48 months of age; and 369 failed to have serum samples collected before (or after) booster immunization and were therefore considered to have voluntarily withdrawn; the remaining 439 eligible children enrolled in the booster immunization clinical trial 201518502-C (bOPV-PRO-C) (Fig. 1, Supplementary Table 1). The baseline demographic characteristics of the participants in each group were similar, with no statistical differences in age, sex, or ethnicity (Supplementary Table 2). After completion of the basic three-dose polio immunization^6^, the positivity rates PV1- and PV3-specific antibody reached or were close to 100% and the PV1 and PV3 antibody GMTs reached high levels in all groups, but the positivity rate for PV2-specific antibody and the GMT induced by the 1IPV + 2bOPV immunization program did not reach the ideal level; additionally, some subjects lacked detectable PV2-specific antibody at 28 days after completion of the basic polio immunization (Supplementary Table 3, Supplementary Table 4).

### Proportion of subjects re-vaccinated with IPV and changes in poliovirus neutralizing antibody levels after vaccination

**Fig. 2:**
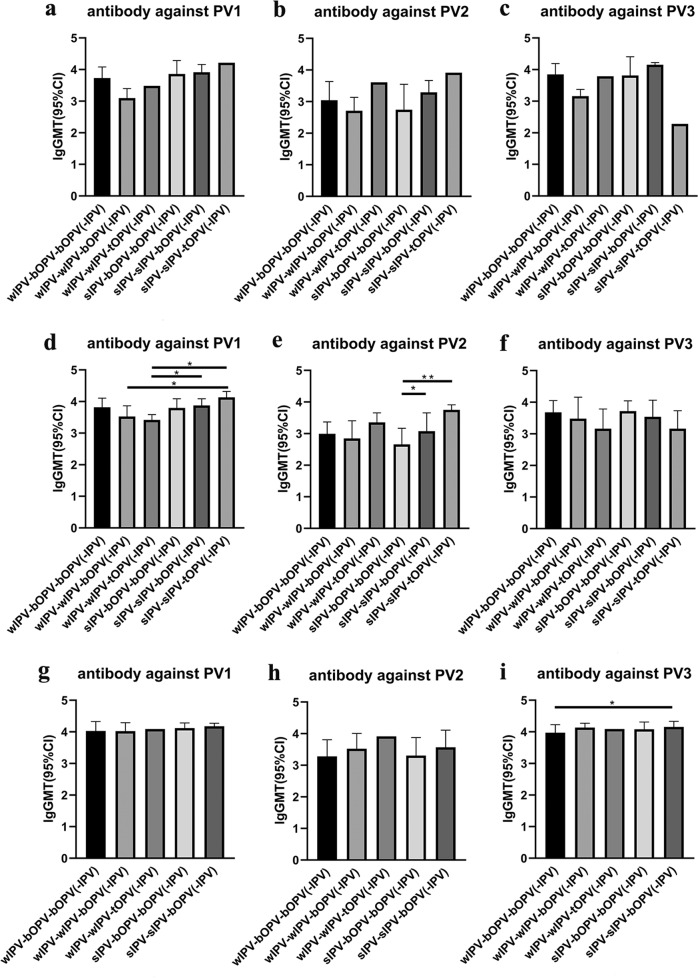
GMT for neutralizing antibody against PV1, PV2, and PV3 at 28 days after revaccination with IPV. Differences in antibody levels between groups were compared after log-transformation of the GMT (95%CI). The sample size is 1 (*n* = 1), so GMT and 95%CI cannot be calculated. Statistical test: Kruskal–Wallis test; *p < 0.05; **p < 0.001. error bars: Standard Deviation (s.d.). **a**–**c** Re-vaccined with IPV at 24 months of age. wIPV-bOPV-bOPV(-IPV): *n* = 28; wIPV-wIPV-bOPV(-IPV): *n* = 2; wIPV-wIPV-tOPV(-IPV): *n* = 1; sIPV-bOPV-bOPV(-IPV): *n* = 49; sIPV-sIPV-bOPV(-IPV): *n* = 4; sIPV-sIPV-tOPV(-IPV): *n* = 1. **d**–**f** Re-vaccined with IPV at 36 months of age. wIPV-bOPV-bOPV(-IPV): *n* = 24; wIPV-wIPV-bOPV(-IPV): *n* = 12; wIPV-wIPV-tOPV(-IPV): *n* = 5; sIPV-bOPV-bOPV(-IPV): *n* = 53; sIPV-sIPV-bOPV(-IPV): *n* = 23; sIPV-sIPV-tOPV(-IPV): *n* = 5. **g**–**i** Re-vaccined with IPV at 48 months of age. wIPV-bOPV-bOPV(-IPV): *n* = 18; wIPV-wIPV-bOPV(-IPV): *n* = 23; wIPV-wIPV-tOPV(-IPV): *n* = 1; sIPV-bOPV-bOPV(-IPV): *n* = 27; sIPV-sIPV-bOPV(-IPV): *n* = 20; sIPV-sIPV-tOPV(-IPV): *n* = 0.

The number of participants who were re-vaccinated with IPV at 24, 36, or 48 months of age varied significantly among the different sequential immunization schedule groups (*P* < 0.0001, *Fisher’s exact test*) (Table 1). Significantly fewer people who had received tOPV or two doses of IPV in their original polio immunization needed to be re-vaccinated with IPV compared with people who received two doses of bOPV in their original immunization. Among those whose original polio immunization schedule was 2IPV + 1bOPV, regardless of whether sIPV or wild/conventional-based IPV (wIPV) was used for basic immunization, no more than 15% or 26% were negative for poliovirus-specific antibody at 36 or 48 months of age, respectively. In contrast, the choice of sIPV vs wIPV did affect the induction of poliovirus-specific antibody when the original polio immunization schedule was 1IPV + 2bOPV. Specifically, after sIPV-bOPV-bOPV basic immunization, 26.1% and 28.2% of participants required revaccination with IPV at 24 and 36 months of age, respectively; thus, without the intervention of revaccination, the number of poliovirus-specific antibody-negative participants at 36 months of age would be >50%. And in the absence of revaccination, basic immunization with wIPV-bOPV-bOPV would result in nearly 30% of subjects being poliovirus-specific antibody negative at 36 months of age (Table 1, Supplementary Table 5). Notably, among those re-vaccinated with IPV, we observed almost 100% antibody positivity for PV1, PV2, and PV3, with high GMTs for antibodies against all three types of poliovirus (Table 2, Fig. 2 and Supplementary Table 6).

**Table 1:** Proportion of study participants re-vaccinated with IPV.

	wIPV-bOPV-bOPV	wIPV-wIPV-bOPV	wIPV-wIPV-tOPV	sIPV-bOPV-bOPV	sIPV-sIPV-bOPV	sIPV-sIPV-tOPV	*P* Value^a^
	*n* = 183	*n* = 183	*n* = 184	*n* = 188	*n* = 186	*n* = 180	
24 months-IPV, *n*(%)	28/183(15.3%)	2/183(1.1%)	1/184(0.5%)	49/188(26.1%)	4/186(2.2%)	1/180(0.6%)	<0.0001
36 months-IPV, *n*(%)	24/183(13.1%)	12/183(6.6%)	5/184(2.7%)	53/188(28.2%)	23/186(12.4%)	5/180(2.8%)	<0.0001
48 months-IPV, *n*(%)	18/183(9.8%)	23/183(12.6%)	1/184(0.5%)	27/188(14.4%)	20/186(10.8%)	0/180(0.0%)	<0.0001

*wIPV* wild/conventional inactivated poliovirus vaccine, *sIPV* Sabin strain-based inactivated poliovirus vaccine, *tOPV* trivalent oral poliovirus vaccine, *bOPV* bivalent oral poliovirus vaccine.

^a^Fisher’s exact test.

**Table 2:** Seropositivity rates for neutralizing antibody against PV1, PV2, and PV3 at 28 days after revaccination with IPV.

	wIPV-bOPV-bOPV(-IPV)	wIPV-wIPV-bOPV(-IPV)	wIPV-wIPV-tOPV(-IPV)	sIPV-bOPV-bOPV(-IPV)	sIPV-sIPV-bOPV(-IPV)	sIPV-sIPV-tOPV(-IPV)	*P* Value^a^
24 months-IPV, n	28	2	1	49	4	1	
Type I							
Seropositivity	28/28(100%)	2/2(100%)	1/1(100%)	49/49(100%)	4/4(100%)	1/1(100%)	1
Type II							
Seropositivity	28/28(100%)	2/2(100%)	1/1(100%)	46/49(93.9%)	4/4(100%)	1/1(100%)	0.479
Type III							
Seropositivity	28/28(100%)	2/2(100%)	1/1(100%)	48/49(98.0%)	4/4(100%)	1/1(100%)	1
36 months-IPV, n	24	12	5	53	23	5	
Type I							
Seropositivity	24/24(100%)	12/12(100%)	5/5(100%)	53/53(100%)	23/23(100%)	5/5(100%)	1
Type II							
Seropositivity	24/24(100%)	12/12(100%)	5/5(100%)	53/53(100%)	23/23(100%)	5/5(100%)	1
Type III							
Seropositivity	24/24(100%)	12/12(100%)	5/5(100%)	53/53(100%)	23/23(100%)	5/5(100%)	1
48 months-IPV, n	18	23	1	27	20	0	
Type I							
Seropositivity	18/18(100%)	23/23(100%)	1/1(100%)	27/27(100%)	20/20(100%)		1
Type II							
Seropositivity	18/18(100%)	23/23(100%)	1/1(100%)	27/27(100%)	20/20(100%)		1
Type III							
Seropositivity	18/18(100%)	23/23(100%)	1/1(100%)	27/27(100%)	20/20(100%)		1

*wIPV* wild/conventional inactivated poliovirus vaccine, *sIPV* Sabin strain-based inactivated poliovirus vaccine, *tOPV* trivalent oral poliovirus vaccine, *bOPV* bivalent oral poliovirus vaccine, *GMT* geometric mean titer, *CI* confidence interval.

^a^Fisher’s exact test.

### Positive rates and titers for poliovirus neutralizing antibody induced by bOPV booster immunization at 48 months of age

As shown in Fig. 1, 439 participants were vaccinated with one dose of bOPV as a booster immunization at 48 months of age, and serum samples were collected from them 28 days later for use in poliovirus neutralizing antibody testing to analyze the changes in poliovirus-specific antibody levels.

Before the bOPV booster immunization at 48 months of age, PV1- and PV3-specific antibody positivity rates were close to 100% in all groups, with no significant difference between groups (PV1: *P* = 1; PV3: *P* = 0.633, *Fisher’s exact test*), and both PV1- and PV3-specific antibody positivity rates reached 100% after bOPV booster immunization at 48 months of age. In contrast, there was a significant difference in the PV2-specific antibody positivity rates before booster immunization at 48 months of age among groups (*P* < 0.0001, Fisher’s exact test); specifically, the 1IPV + 2bOPV groups had lower rates than 2IPV + 1bOPV groups (Table 3, Supplementary Table 7). However, after the administration of one dose of bOPV as a booster immunization at 48 months of age, the PV2-specific antibody positivity rate was at or near 100% in all groups and there was no longer a significant difference between groups (*P* = 0.177, Fisher’s exact test) (Table 3).

**Table 3:** Seropositivity rates for neutralizing antibody against PV1, PV2, and PV3 before and after booster immunization at 48 months of age.

	wIPV-bOPV-bOPV(-bOPV)	wIPV-wIPV-bOPV(-bOPV)	wIPV-wIPV-tOPV(-bOPV)	sIPV-bOPV-bOPV(-bOPV)	sIPV-sIPV-bOPV(-bOPV)	sIPV-sIPV-tOPV(-bOPV)	*P* Value^a^
	*n* = 58	*n* = 73	*n* = 109	*n* = 20	*n* = 71	*n* = 108	
48 months							
Type 1							
Seropositivity, n(%)	58/58(100%)	73/73(100%)	108/109(99.1%)	20/20(100%)	71/71(100%)	108/108(100%)	1
Type 2							
Seropositivity, n(%)	55/58(94.8%)	72/73(98.6%)	109/109(100%)	18/20(90%)	65/71(91.5%)	108/108(100%)	<0.0001
Type 3							
Seropositivity, n(%)	58/58(100%)	73/73(100%)	109/109(100%)	20/20(100%)	70/71(98.6%)	107/108(99.1%)	0.633
48 months-bOPV							
Type 1							
Seropositivity, n(%)	58/58(100%)	73/73(100%)	109/109(100%)	20/20(100%)	71/71(100%)	108/108(100%)	1
Type 2							
Seropositivity, n(%)	57/58(98.3%)	73/73(100%)	109/109(100%)	20/20(100%)	71/71(100%)	108/108(100%)	0.177
Type 3							
Seropositivity, n(%)	58/58(100%)	73/73(100%)	109/109(100%)	20/20(100%)	71/71(100%)	108/108(100%)	1

*wIPV* wild/conventional inactivated poliovirus vaccine, *sIPV* Sabin strain-based inactivated poliovirus vaccine, *tOPV* trivalent oral poliovirus vaccine, *bOPV* bivalent oral poliovirus vaccine, *GMT* geometric mean titer, *CI* confidence interval.

^a^Fisher’s exact test.

**Fig. 3:**
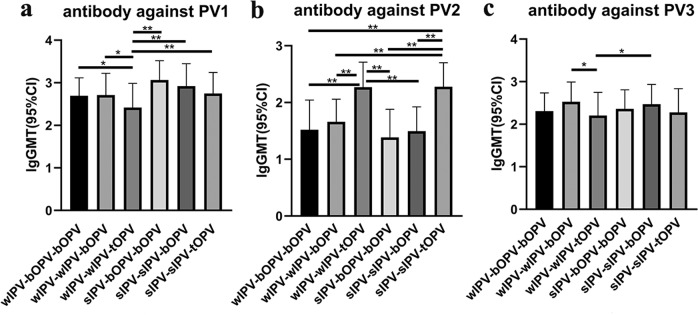
GMT for neutralizing antibody against PV1, PV2, and PV3 before booster immunization at 48 months of age. Differences in antibody levels between groups were compared after log-transformation of the GMT (95%CI). Statistical test: Kruskal–Wallis test; *p < 0.05; **p < 0.001. error bars: Standard Deviation (s.d.). **a** GMT for neutralizing antibody against PV1. **b** GMT for neutralizing antibody against PV2. **c** GMT for neutralizing antibody against PV3. wIPV-bOPV-bOPV: *n* = 58; wIPV-wIPV-bOPV: *n* = 73; wIPV-wIPV-tOPV: *n* = 109; sIPV-bOPV-bOPV: *n* = 20; sIPV-sIPV-bOPV: *n* = 71; sIPV-sIPV-tOPV: *n* = 108.

**Fig. 4:**
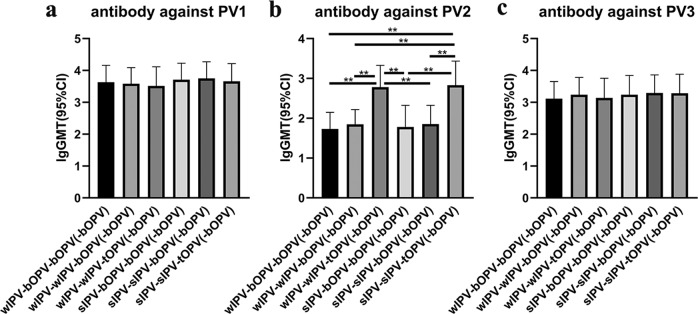
GMT for neutralizing antibody against PV1, PV2, and PV3 at 28 days after booster immunization at 48 months of age. Differences in antibody levels between groups were compared after log-transformation of the GMT (95%CI). Statistical test: Kruskal–Wallis test; *p < 0.05; **p < 0.001. error bars: Standard Deviation (s.d.). **a** GMT for neutralizing antibody against PV1. **b** GMT for neutralizing antibody against PV2. **c** GMT for neutralizing antibody against PV3. wIPV-bOPV-bOPV(-bOPV): *n* = 58; wIPV-wIPV-bOPV(-bOPV): *n* = 73; wIPV-wIPV-tOPV(-bOPV): *n* = 109; sIPV-bOPV-bOPV(-bOPV): *n* = 20; sIPV-sIPV-bOPV(-bOPV): *n* = 71; sIPV-sIPV-tOPV(-bOPV): *n* = 108.

Before the bOPV booster immunization at 48 months of age, the GMTs of PV1-, PV2-, and PV3-specific antibodies, were significantly different in all groups (*P* < 0.0001; *P* < 0.0001; *P* = 0.0002, respectively, Kruskal–Wallis test). Under matched sequential immunization programs, better immunopersistence of PV1-antibodies was induced by using sIPV than by using wIPV, and the 2IPV + 1bOPV programs induced higher titers of PV2-specific antibody compared with the programs that used 1IPV + 2bOPV. The polio immunization schedule with two doses of IPV induced higher titers of PV2-specific antibody than did the schedule with only one dose of IPV (Fig. 3, Supplementary Table 8). After the booster immunization with one dose of bOPV at 48 months of age, the GMTs of PV1- and PV3-specific antibodies were not significantly different between the groups (PV1, *P* = 0.0525; PV3, *P* = 0.2431, Kruskal–Wallis test), but the GMTs of PV2-specific antibody were still significantly different between groups (*P* < 0.0001, Kruskal–Wallis test). The characteristics of the differences between groups were consistent with those before the booster immunization, with the GMTs of poliovirus-specific antibodies induced by the 2IPV + 2bOPV groups being higher than those induced by the 1IPV + 3bOPV groups (Fig. 4, Supplementary Table 9).

## Discussion

Polio vaccine attenuated virus strains are genetically unstable, and Thompson and Duintjer Tebbens^16^ showed by modeling that a coordinated global cessation of OPV use would be required to maintain a world free of poliovirus-induced poliomyelitis after the eradication of WPV. However, a shortage of IPV^17^ and low coverage for basic polio immunization^18^ exist in some parts of the world owing to insufficient of IPV production, underdeveloped local economy, and religious beliefs. In 2020, some countries and regions in Africa and South America had only approximately 41% of 1-year-old children covered by polio basic immunization^18^. The shift in immunization strategies has resulted in insufficient levels of population immunity and low protection against PV2; consequently, global circulating VDPV type 2 (cVDPV2) cases are on the rise, with an increase in the frequency and geographical distribution of outbreaks worldwide^19^. In 2018, WHO recognized cVDPV2 outbreaks as “public health emergencies of international concern”^20^. In 2015, before the switch in vaccine strategy, only 12 cases caused by cVDPV2 occurred worldwide^21^, and from June 2021 to June 2022, cVDPV2 affected a total of 16 countries and territories, causing a total of 587 cases. In addition to cVDPV2, there were 12 cases caused by type 1-VDPV in Madagascar and one case caused by type 3-VDPV in Israel^22^. On July 22, 2022, the New York State Department of Health in the United States reported a confirmed case of polio in which the patient was infected with a virus consistent with the Sabin type 2 vaccine strain according to genetic sequencing results^23^. The problem caused by the switch of polio immunization strategies has given rise to new challenges for the global polio eradication campaign.

To optimize polio sequential immunization programs, clinical studies of basic and booster immunization are very important, with particular attention to the polio immunization status in developing countries that use sIPV for sequential immunization. Our findings may provide supportive evidence for booster immunization to the sequential polio vaccine immunization schedule. For using of 1IPV + 2bOPV as a basic immunization program, if the goal is that no less than 70% of polio vaccine recipients have persistent protective efficacy against all three types of polioviruses (especially PV2), our data on the percentage of participants who required revaccination with IPV (Table 1) support a booster immunization with IPV at 36 months of age, particularly after the use of sIPV. A controlled clinical trial conducted in Pakistan^24^ also showed that bOPV induced seroconversion against PV2, but the rate of seroconversion was low, with only 18% of seroconversion against PV2 induced in the group immunized with four doses of bOPV. The effectiveness of the immune barrier against WPV or VDPV infection depends largely on the level of neutralizing antibodies in an individual’s serum^1,25^.

However, at this stage, the global IPV supply is still limited with a large demand; consequently, some regions can guarantee a sequential immunization program containing only one dose of IPV. According to the results of the present study, whether wIPV or sIPV is used, a regimen of 1IPV + 3bOPV results in a relatively weak antibody response to PV2, and the induced antibodies do not persist for as long, potentially increasing the risk of infection with cVDPV2. Only by including more than two doses of IPV sequential immunization can sufficient levels of PV2 neutralizing antibodies be generated and better immune persistence against PV2 be obtained. WHO made clear through a position paper to ensure the WHO position paper (June 2022)^26^ to ensure a sequential immunization program of at least 2 doses of IPV. Our IPV revaccination results show that IPV booster vaccination at suitable time for different sequential immunization programs also induced high levels of antibodies against all three types of polioviruses, particularly important for those who have vaccinated only 1 dose of IPV as part of their basic immunization. Therefore, to counteract the risk posed by cVDPV2, there is a need to add a PV2 component to booster immunization via the use of IPV or the novel serotype 2 oral live attenuated polio vaccine (nOPV2)^27^. This new polio vaccine strain based on the Sabin 2 vaccine strain was better genetic stability and safer^28–31^. Currently, two polio vaccine candidate type 2 strains are in the clinical testing phase^32,33^.

A phase III clinical trial conducted by Hu et al. ^34,35^ showed that sIPV has an immunogenicity profile that is not inferior to that of wIPV and has a good safety profile. Additionally, a phase IV clinical trial conducted by Yan^36^ et al. found that, regarding the seroconversion rate for antibodies against PV1, PV2, and PV3, the 2sIPV + 1bOPV group was not inferior to the 3sIPV group; however, for the seroconversion rate of anti-PV2 antibody, the 1sIPV + 2bOPV group was inferior to the 3sIPV group. Because the production of sIPV using the Sabin strain has lower biosafety risks and production costs, it is suitable for production and use in developing countries, which has led to an expanded manufacturing base for IPV^37^. An increase in IPV production would make it possible to gradually increase the number of IPV doses in the polio immunization schedule or use IPV for booster immunization and to gradually convert to using a full IPV immunization schedule as soon as possible after the global elimination of wild strains to reduce the occurrence of VDPV.

In addition to VDPV, WPV type 1 (WPV1) is still endemic in a few countries, such as Pakistan, Afghanistan, Malawi, and Mozambique^22^. The recent WPV1 case reported in Malawi is the first WPV1 case since the African region was declared free of WPV, and the strain is genetically related to the strain spectrum detected in Pakistan in 2019^38^. Another case of WPV1 was subsequently detected in Mozambique, which shares a border with Malawi, in May 2022^22^. Consequently, other locations adjacent to these countries, such as China, are at high risk for polio importation. Until the transmission of WPV1 is blocked, other non-adjacent countries will also be at risk for polio importation. Compared with the sequential immunization schedule of 2IPV + 2bOPV or 3IPV + 1bOPV, the alternating sequential immunization schedule of 2IPV + 1bOPV + 1IPV can safely ensure the induction of high antibody levels against all three types of polioviruses while allowing an earlier induction of intestinal mucosal immunity, which is conducive to polio control and prevention in areas with WPV and VDPV transmission or high import risk, and therefore help prevent WPV and VDPV transmission and outbreaks.

In summary, to protect children from VDPV2, the addition of a PV2 vaccine component to booster immunization or the use of IPV for booster immunization should be considered. For children who received only one dose of IPV during the basic immunization phase, to ensure that no less than 70% of them have effective protection, our data support the administration of a booster immunization at 36 months old, ideally with IPV. Furthermore, especially in WPV and VDPV2 high-risk areas, an alternating sequential immunization schedule of 2IPV + 1bOPV + 1IPV is recommended to ensure that the immunization program can induce some intestinal mucosal immunity and high levels of antibody against PV1 and PV3 and to increase induced immunity against PV2 in the safe way possible. It can effectively prevent and control the occurrence of WPV, vaccine-associated paralytic poliomyelitis, and vaccine-derived poliovirus.

## Methods

### Study design

This booster immunization clinical trial 201518502-C (bOPV-PRO-C) (Clinical Trials.gov number: NCT03821441) to evaluate booster immunization timing and effects was expanded from the original completed phase III clinical trial “Randomized, Double Blind, Single Center, Parallel Trial to Evaluate the Safety and Immunogenicity By Different Sequential Immunization Schedules of Bivalent Oral Poliomyelitis Vaccine Co-administered With IPV in Infants Aged 2 Months”. The present trial (NCT03821441), launched in 2018 and completed in 2020, was sponsored by the Institute of Medical Biology, Chinese Academy of Medical Sciences (IMBCAMS) and conducted by the Guangxi Zhuang Autonomous Region Center for Disease Control and Prevention, with clinical trial sites in Liujiang District, Liucheng County, and Rong’an County, Liuzhou City, Guangxi, China. The study was approved by the Ethics Committee of the Guangxi Zhuang Autonomous Region Center for Disease Control and Prevention (approval number: GXIRB2017-0009-2) and was conducted in accordance with the Declaration of Helsinki (revised 2013). Informed consent was obtained in writing from the legal guardians of all study participants.

### Participants

The inclusion criteria were as follows: (1) prior participation in phase III clinical trial in Guangxi, with completion of three-dose primary polio immunization and available paired serum results; (2) age of 24 months old (calendar month); (3) voluntary informed consent provided by their guardian; and (4) able to attend all scheduled visits and to comply with all trial procedures (including vaccination and blood collection). The exclusion criteria were as follows: (1) any booster immunization with polio vaccine after finishing participation in the prior three-dose primary immunization research; (2) poliovirus infection as demonstrated with laboratory experiment; (3) participation in another concurrent clinical trial; and (4) any condition that, in the opinion of the investigator, may interfere with the evaluation of study objectives or increase the risk to the potential subject, such as acute or chronic diseases or a laboratory-detected abnormality.

The guardians and families of the participants voluntarily complied with the requirements of the clinical trial protocol. An informed consent form was signed by both the guardians and the study doctor of each participant prior to initiation of the clinical trial. Participants were permitted to withdraw voluntarily at any time during the trial. Participants could be withdrawn from the study in cases of failure to adhere to the follow-up visits, violation of or deviation from the trial protocol, or the appearance of other abnormal symptoms that interfered with the trial.

### Vaccines

The following vaccines, also used in the prior phase III clinical trial, were used in the present clinical trial (Clinical Trials.gov number: 201518502-C (bOPV-PRO-C)): (1) bOPV (Candy), a bivalent oral attenuated live poliomyelitis vaccine against PV1 and PV3 in Dragee Candy (human diploid cells) produced by IMBCAMS, available in 1-g pills (10 pills/patch), administered in one-pill doses with each pill containing ≥5.92 lgCCID_50_ of poliovirus, including ≥5.8 lgCCID_50_ of PV1 and ≥5.3 lgCCID_50_ of PV3; (2) bOPV (Liquid), a bivalent oral attenuated live poliomyelitis vaccine against PV1 and PV3 (human diploid cells) produced by Beijing Tiantan Biological Products Co., Ltd. available in 1.0-ml bottles, administered in 2-drop doses each person (equivalent to 0.1 ml of vaccine per person) containing ≥6.12 lgCCID_50_ of total poliovirus, including ≥6.0 lgCCID_50_ of PV1 and ≥5.5 lgCCID_50_ of PV3; and (3) sIPV, a Sabin strain-based inactivated poliovirus vaccine provided by IMBCAMS for which each dose (0.5 ml) contained 30, 32, and 45 D-antigen units of PV1, PV2, and PV3, respectively.

### Procedures

On the basis of the original phase III clinical trial, venous blood was collected at ages 24, 36, and 48 months from all study participants, and serum was isolated for the determination of PV1-, PV2- and PV3-neutralizing antibody titers. In the groups of subjects who received only one dose of IPV (either wIPV or sIPV) during the basic immunization phase, there were low anti-PV2 antibody positivity rates, and some participants had no detectable anti-PV2 antibody; additionally, a very small number of these subjects had no detectable antibodies against PV1 and PV3. Therefore, in accordance with ethical requirements and trial design, study participants found to be negative for neutralizing antibodies against PV1, PV2, or PV3 (neutralizing antibody titer of <1:8) at 24, 36, 48 months of age were promptly re-vaccinated with sIPV. Blood was collected from these participants 28 days after this additional vaccination to determine the poliovirus neutralizing antibody titers and calculate the post-revaccination poliovirus neutralizing antibody positivity rates and GMTs. The study participants who had not been re-vaccinated with sIPV at 24, 36, or 48 months of age were given one dose of bOPV as a booster immunization after the completion of blood collection at 48 months of age, and venous blood was collected from these participants for antibody testing 28 days after booster immunization.

The 439 participants in this clinical trial were divided into the following six groups according to the basic polio immunization schedule they had received in the original phase III trial: (1) wIPV-bOPV-bOPV(-bOPV); (2) wIPV-wIPV-bOPV(-bOPV); (3) wIPV-wIPV-tOPV(-bOPV); (4) sIPV-bOPV-bOPV(-bOPV); (5) sIPV-sIPV-bOPV(-bOPV); and (6) sIPV-sIPV-tOPV(-bOPV). For analysis, these groups were sometimes categorized into larger groups of participants that received two doses of IPV and one dose of bOPV (2IPV + 1bOPV; groups 2 and 5), received one dose of IPV and two doses of bOPV (1IPV + 2bOPV; groups 1 and 4), or received any doses of tOPV.

After they received a dose of polio vaccine, participants were observed for 30 min in accordance with the approved protocol. Any adverse events that occurred within 30 days after vaccination were recorded. Antibody titers were measured using the micro-neutralization test with Sabin strains by the National Institutes for Food and Drug Control in accordance with the protocol recommended by WHO^39^. Seropositivity was defined as having a neutralizing antibody titer ≥ 1:8 after receiving a booster or revaccination. Changes in antibody titers after vaccination were analyzed. The maximum dilution and the maximum reported titer were both 16384; in the case of an actual titer being greater than 16384, the value used in our calculations was 16384; in the case of an actual titer being less than 8, the value used in our calculations was 4.

### Statistical analysis

Positivity rates for serum antibody against PV1, PV2, and PV3 were calculated, and the difference in antibody-positive rates between groups was compared by conducting a Fisher’s exact test. Differences in antibody titers between groups were compared by performing a one-way ANOVA or Kruskal–Wallis nonparametric test after logarithmic-function transformation (logarithm of GMT with a base of 10) of the data. Two-sided values of *P* < 0.05 were considered statistically significant.

### Reporting summary

Further information on research design is available in the Nature Research Reporting Summary linked to this article.

## Supplementary information

Supplementary Info

REPORTING SUMMARY

## Data Availability

The data that support the findings of this study are available from the corresponding author upon reasonable request. The booster immunization clinical trial 201518502-C (bOPV-PRO-C)(Clinical Trials.gov number: NCT03821441) can be found in clinicaltrials.gov (https://clinicaltrials.gov/ct2/home).
