# Supplementary material for: Evaluation of the immunization effectiveness of bOPV booster immunization and IPV revaccination
Source: NPJ Vaccines. 2023 Mar 18;8:44. doi: 10.1038/s41541-023-00642-w (PMC10024706; doi:10.1038/s41541-023-00642-w)
Supplement: Supplementary file 1 — Supplementary Info [file 41541_2023_642_MOESM1_ESM.pdf]

Supplementary

Supplementary Table 1 Explanation table for the number of subjects enrolled bOPV booster immunization

	wIPV-bOPV-bOPV(-bOPV)	wIPV-wIPV-bOPV(-bOPV)	wIPV-wIPV-tOPV(-bOPV)	sIPV-bOPV-bOPV(-bOPV)	sIPV-sIPV-bOPV(-bOPV)	sIPV-sIPV-tOPV(-bOPV)
	n=183	n=183	n=184	n=188	n=186	n=180
24 months-IPV, n	28	2	1	49	4	1
36 months-IPV, n	24	12	5	53	23	5
48 months-IPV, n	18	23	1	27	20	0
Samples dropped out, n	55	73	68	39	68	66
Enrolled the booster immunization clinical trial, n	58	73	109	20	71	108

wIPV：conventional inactivated poliovirus vaccine；sIPV：Sabin strain-based inactivated poliovirus vaccine；tOPV：trivalent oral poliovirus vaccine；bOPV：bivalent oral poliovirus vaccine

Supplementary Table 2 Demographic characteristics of Subjects

	wIPV-bOPV-bOPV	wIPV-wIPV-bOPV	wIPV-wIPV-tOPV	sIPV-bOPV-bOPV	sIPV-sIPV-bOPV	sIPV-sIPV-tOPV	*P* Value
	(n=183)	(n=183)	(n=184)	(n=188)	(n=186)	(n=180)	
Age (years), mean ± SD	2.6±0.1	2.6±0.1	2.6±0.1	2.6±0.1	2.6±0.1	2.6±0.1	0.99[a]
Sex, n (%)							0.46[b]
Male	90(49.2%)	105(57.4%)	97(52.7%)	107(56.9%)	100(53.8%)	89(49.4%)	
Female	93(50.8%)	78(42.6%)	87(47.3%)	81(43.1%)	86(46.2%)	91(50.6%)	
Race, n (%)							
Han	53(33%)	59(32.2%)	62(33.7%)	62(33%)	50(26.9%)	48(26.7%)	0.5[b]
Zhuang	115(62.8%)	111(60.7%)	106(57.6%)	113(60.1%)	121(65.1%)	116(64.4%)	0.7[b]
Miao	4(2.2%)	4(2.2%)	5(2.7%)	4(2.1%)	4(2.2%)	3(1.7%)	1[b]
Yao	1(0.5%)	3(1.6%)	0(0%)	1(0.5%)	1(0.5%)	2(1.1%)	0.5[b]
Other	10(5.5%)	6(3.3%)	11(6%)	8(4.3%)	10(5.4%)	11(6.1%)	0.8[b]

wIPV：conventional inactivated poliovirus vaccine；sIPV：Sabin strain-based inactivated poliovirus vaccine；tOPV：trivalent oral poliovirus vaccine；bOPV：bivalent oral poliovirus vaccine. SD: standard deviation. a: Kruskal-Wallis test. b: Pearson’s chi-square test.

Supplementary Table 3 Negative rate for neutralizing antibody against PV1, PV2, and PV3 at 28 days after basic immunization

	wIPV-bOPV-bOPV	wIPV-wIPV-bOPV	wIPV-wIPV-tOPV	sIPV-bOPV-bOPV	sIPV-sIPV-bOPV	sIPV-sIPV-tOPV	*P* Value
	n=194	n=194	n=191	n=195	n=197	n=194	
Type 1							
Negative , n(%)	0/194（0%)	0/194（0%)	0/191（0%)	0/195（0%)	0/197（0%)	0/194（0%)	1[a]
Type 2							
Negative , n(%)	34/194（17.5%)	2/194（1.0%)	1/191（0.5%)	55/195（28.2%)	5/197（2.5%)	0/194（0%)	<0.0001[a]
Type 3							
Negative , n(%)	0/194（0%)	0/194（0%)	0/191（0%)	0/195（0%)	0/197（0%)	1/194（0.5%)	0.664[a]

wIPV：wild/conventional inactivated poliovirus vaccine；sIPV：Sabin strain-based inactivated poliovirus vaccine；tOPV：trivalent oral poliovirus vaccine；bOPV：bivalent oral poliovirus vaccine. a: Fisher’s exact test.

Supplementary Table 4 Multiple comparisons of seronegativity against PV2 at 28 days after basic immunization

Immunogenicity indicator	Group	VS.	Group	Test method	*P* value
poliovirus type 2	wIPV-bOPV-bOPV	VS.	wIPV-wIPV-bOPV	Fisher exact test	<0.0001
			wIPV-wIPV-tOPV	Fisher exact test	<0.0001
			sIPV-bOPV-bOPV	Fisher exact test	0.016
			sIPV-sIPV-bOPV	Fisher exact test	<0.0001
			sIPV-sIPV-tOPV	Fisher exact test	<0.0001
	wIPV-wIPV-bOPV	VS.	wIPV-wIPV-tOPV	Fisher exact test	1
			sIPV-bOPV-bOPV	Fisher exact test	<0.0001
			sIPV-sIPV-bOPV	Fisher exact test	0.449
			sIPV-sIPV-tOPV	Fisher exact test	0.499
	wIPV-wIPV-tOPV	VS.	sIPV-bOPV-bOPV	Fisher exact test	<0.0001
			sIPV-sIPV-bOPV	Fisher exact test	0.215
			sIPV-sIPV-tOPV	Fisher exact test	0.496
	sIPV-bOPV-bOPV	VS.	sIPV-sIPV-bOPV	Fisher exact test	<0.0001
			sIPV-sIPV-tOPV	Fisher exact test	<0.0001
	sIPV-sIPV-bOPV	VS.	sIPV-sIPV-tOPV	Fisher exact test	0.061

wIPV：conventional inactivated poliovirus vaccine；sIPV：Sabin strain-based inactivated poliovirus vaccine；tOPV：trivalent oral poliovirus vaccine；bOPV：bivalent oral poliovirus vaccine.

Supplementary Table 5 Multiple comparisons of the number of subjects re-vaccinated with 1 dose of IPV

age of revaccination	Group	VS.	Group	Test method	*P* value
24 months of age	wIPV-bOPV-bOPV	VS.	wIPV-wIPV-bOPV	Fisher exact test	<0.0001
			wIPV-wIPV-tOPV	Fisher exact test	<0.0001
			sIPV-bOPV-bOPV	Fisher exact test	0.015
			sIPV-sIPV-bOPV	Fisher exact test	<0.0001
			sIPV-sIPV-tOPV	Fisher exact test	<0.0001
	wIPV-wIPV-bOPV	VS.	wIPV-wIPV-tOPV	Fisher exact test	0.623
			sIPV-bOPV-bOPV	Fisher exact test	<0.0001
			sIPV-sIPV-bOPV	Fisher exact test	0.685
			sIPV-sIPV-tOPV	Fisher exact test	1
	wIPV-wIPV-tOPV	VS.	sIPV-bOPV-bOPV	Fisher exact test	<0.0001
			sIPV-sIPV-bOPV	Fisher exact test	0.372
			sIPV-sIPV-tOPV	Fisher exact test	1
	sIPV-bOPV-bOPV	VS.	sIPV-sIPV-bOPV	Fisher exact test	<0.0001
			sIPV-sIPV-tOPV	Fisher exact test	<0.0001
	sIPV-sIPV-bOPV	VS.	sIPV-sIPV-tOPV	Fisher exact test	0.373
36 months of age	wIPV-bOPV-bOPV	VS.	wIPV-wIPV-bOPV	Fisher exact test	0.052
			wIPV-wIPV-tOPV	Fisher exact test	0.00018
			sIPV-bOPV-bOPV	Fisher exact test	0.00049
			sIPV-sIPV-bOPV	Fisher exact test	0.877
			sIPV-sIPV-tOPV	Fisher exact test	0.00033
	wIPV-wIPV-bOPV	VS.	wIPV-wIPV-tOPV	Fisher exact test	0.088
			sIPV-bOPV-bOPV	Fisher exact test	<0.0001
			sIPV-sIPV-bOPV	Fisher exact test	0.075
			sIPV-sIPV-tOPV	Fisher exact test	0.134

	wIPV-wIPV-tOPV	VS.	sIPV-bOPV-bOPV	Fisher exact test	<0.0001
			sIPV-sIPV-bOPV	Fisher exact test	0.001
			sIPV-sIPV-tOPV	Fisher exact test	1
	sIPV-bOPV-bOPV	VS.	sIPV-sIPV-bOPV	Fisher exact test	0.00017
			sIPV-sIPV-tOPV	Fisher exact test	<0.0001
	sIPV-sIPV-bOPV	VS.	sIPV-sIPV-tOPV	Fisher exact test	0.001
48 months of age	wIPV-bOPV-bOPV	VS.	wIPV-wIPV-bOPV	Fisher exact test	0.508
			wIPV-wIPV-tOPV	Fisher exact test	0.00003
			sIPV-bOPV-bOPV	Fisher exact test	0.205
			sIPV-sIPV-bOPV	Fisher exact test	1
			sIPV-sIPV-tOPV	Fisher exact test	<0.0001
	wIPV-wIPV-bOPV	VS.	wIPV-wIPV-tOPV	Fisher exact test	<0.0001
			sIPV-bOPV-bOPV	Fisher exact test	0.65
			sIPV-sIPV-bOPV	Fisher exact test	0.515
			sIPV-sIPV-tOPV	Fisher exact test	<0.0001
	wIPV-wIPV-tOPV	VS.	sIPV-bOPV-bOPV	Fisher exact test	<0.0001
			sIPV-sIPV-bOPV	Fisher exact test	0.00003
			sIPV-sIPV-tOPV	Fisher exact test	1
	sIPV-bOPV-bOPV	VS.	sIPV-sIPV-bOPV	Fisher exact test	0.271
			sIPV-sIPV-tOPV	Fisher exact test	<0.0001
	sIPV-sIPV-bOPV	VS.	sIPV-sIPV-tOPV	Fisher exact test	<0.0001

wIPV：conventional inactivated poliovirus vaccine；sIPV：Sabin strain-based inactivated poliovirus vaccine；tOPV：trivalent oral poliovirus vaccine；bOPV：bivalent oral poliovirus vaccine.

Supplementary Table 6 Multiple comparison of neutralizing antibody geometric mean titers (GMT) at 28 days after re-vaccination with IPV

age-Immunogenicity indicator	Group	VS.	Group	Test method	*P* value
24 months-type 1	wIPV-bOPV-bOPV(-IPV)	VS.	wIPV-wIPV-bOPV(-IPV)	Kruskal-Wallis test	>0.9999
			wIPV-wIPV-tOPV(-IPV)	Kruskal-Wallis test	>0.9999
			sIPV-bOPV-bOPV(-IPV)	Kruskal-Wallis test	0.544
			sIPV-sIPV-bOPV(-IPV)	Kruskal-Wallis test	>0.9999
			sIPV-sIPV-tOPV(-IPV)	Kruskal-Wallis test	>0.9999
	wIPV-wIPV-bOPV(-IPV)	VS.	wIPV-wIPV-tOPV(-IPV)	Kruskal-Wallis test	>0.9999
			sIPV-bOPV-bOPV(-IPV)	Kruskal-Wallis test	0.3279
			sIPV-sIPV-bOPV(-IPV)	Kruskal-Wallis test	0.9012
			sIPV-sIPV-tOPV(-IPV)	Kruskal-Wallis test	0.3161
	wIPV-wIPV-tOPV(-IPV)	VS.	sIPV-bOPV-bOPV(-IPV)	Kruskal-Wallis test	>0.9999
			sIPV-sIPV-bOPV(-IPV)	Kruskal-Wallis test	>0.9999
			sIPV-sIPV-tOPV(-IPV)	Kruskal-Wallis test	>0.9999
	sIPV-bOPV-bOPV(-IPV)	VS.	sIPV-sIPV-bOPV(-IPV)	Kruskal-Wallis test	>0.9999
			sIPV-sIPV-tOPV(-IPV)	Kruskal-Wallis test	>0.9999
	sIPV-sIPV-bOPV(-IPV)	VS.	sIPV-sIPV-tOPV(-IPV)	Kruskal-Wallis test	>0.9999
24 months-type 3	wIPV-bOPV-bOPV(-IPV)	VS.	wIPV-wIPV-bOPV(-IPV)	Kruskal-Wallis test	0.8389
			wIPV-wIPV-tOPV(-IPV)	Kruskal-Wallis test	>0.9999
			sIPV-bOPV-bOPV(-IPV)	Kruskal-Wallis test	>0.9999
			sIPV-sIPV-bOPV(-IPV)	Kruskal-Wallis test	0.6798
			sIPV-sIPV-tOPV(-IPV)	Kruskal-Wallis test	>0.9999
	wIPV-wIPV-bOPV(-IPV)	VS.	wIPV-wIPV-tOPV(-IPV)	Kruskal-Wallis test	>0.9999
			sIPV-bOPV-bOPV(-IPV)	Kruskal-Wallis test	0.5901
			sIPV-sIPV-bOPV(-IPV)	Kruskal-Wallis test	0.0654
			sIPV-sIPV-tOPV(-IPV)	Kruskal-Wallis test	>0.9999

	wIPV-wIPV-tOPV(-IPV)	VS.	sIPV-bOPV-bOPV(-IPV)	Kruskal-Wallis test	>0.9999
			sIPV-sIPV-bOPV(-IPV)	Kruskal-Wallis test	>0.9999
			sIPV-sIPV-tOPV(-IPV)	Kruskal-Wallis test	>0.9999
	sIPV-bOPV-bOPV(-IPV)	VS.	sIPV-sIPV-bOPV(-IPV)	Kruskal-Wallis test	0.8822
			sIPV-sIPV-tOPV(-IPV)	Kruskal-Wallis test	>0.9999
	sIPV-sIPV-bOPV(-IPV)	VS.	sIPV-sIPV-tOPV(-IPV)	Kruskal-Wallis test	0.2387
36 months-type 1	wIPV-bOPV-bOPV(-IPV)	VS.	wIPV-wIPV-bOPV(-IPV)	Kruskal-Wallis test	0.1956
			wIPV-wIPV-tOPV(-IPV)	Kruskal-Wallis test	0.0928
			sIPV-bOPV-bOPV(-IPV)	Kruskal-Wallis test	>0.9999
			sIPV-sIPV-bOPV(-IPV)	Kruskal-Wallis test	>0.9999
			sIPV-sIPV-tOPV(-IPV)	Kruskal-Wallis test	0.4391
	wIPV-wIPV-bOPV(-IPV)	VS.	wIPV-wIPV-tOPV(-IPV)	Kruskal-Wallis test	>0.9999
			sIPV-bOPV-bOPV(-IPV)	Kruskal-Wallis test	0.1895
			sIPV-sIPV-bOPV(-IPV)	Kruskal-Wallis test	0.0624
			sIPV-sIPV-tOPV(-IPV)	Kruskal-Wallis test	0.0038
	wIPV-wIPV-tOPV(-IPV)	VS.	sIPV-bOPV-bOPV(-IPV)	Kruskal-Wallis test	0.1026
			sIPV-sIPV-bOPV(-IPV)	Kruskal-Wallis test	0.0383
			sIPV-sIPV-tOPV(-IPV)	Kruskal-Wallis test	0.002
	sIPV-bOPV-bOPV(-IPV)	VS.	sIPV-sIPV-bOPV(-IPV)	Kruskal-Wallis test	>0.9999
			sIPV-sIPV-tOPV(-IPV)	Kruskal-Wallis test	0.2069
	sIPV-sIPV-bOPV(-IPV)	VS.	sIPV-sIPV-tOPV(-IPV)	Kruskal-Wallis test	0.8971
36 months-type 2	wIPV-bOPV-bOPV(-IPV)	VS.	wIPV-wIPV-bOPV(-IPV)	Kruskal-Wallis test	>0.9999
			wIPV-wIPV-tOPV(-IPV)	Kruskal-Wallis test	>0.9999
			sIPV-bOPV-bOPV(-IPV)	Kruskal-Wallis test	0.1628
			sIPV-sIPV-bOPV(-IPV)	Kruskal-Wallis test	>0.9999
			sIPV-sIPV-tOPV(-IPV)	Kruskal-Wallis test	0.1188

	wIPV-wIPV-bOPV(-IPV)	VS.	wIPV-wIPV-tOPV(-IPV)	Kruskal-Wallis test	>0.9999
			sIPV-bOPV-bOPV(-IPV)	Kruskal-Wallis test	>0.9999
			sIPV-sIPV-bOPV(-IPV)	Kruskal-Wallis test	>0.9999
			sIPV-sIPV-tOPV(-IPV)	Kruskal-Wallis test	0.0722
	wIPV-wIPV-tOPV(-IPV)	VS.	sIPV-bOPV-bOPV(-IPV)	Kruskal-Wallis test	0.0587
			sIPV-sIPV-bOPV(-IPV)	Kruskal-Wallis test	>0.9999
			sIPV-sIPV-tOPV(-IPV)	Kruskal-Wallis test	>0.9999
	sIPV-bOPV-bOPV(-IPV)	VS.	sIPV-sIPV-bOPV(-IPV)	Kruskal-Wallis test	0.0233
			sIPV-sIPV-tOPV(-IPV)	Kruskal-Wallis test	0.0005
	sIPV-sIPV-bOPV(-IPV)	VS.	sIPV-sIPV-tOPV(-IPV)	Kruskal-Wallis test	0.3096
48 months-type 3	wIPV-bOPV-bOPV(-IPV)	VS.	wIPV-wIPV-bOPV(-IPV)	Kruskal-Wallis test	0.3089
			wIPV-wIPV-tOPV(-IPV)	Kruskal-Wallis test	>0.9999
			sIPV-bOPV-bOPV(-IPV)	Kruskal-Wallis test	0.687
			sIPV-sIPV-bOPV(-IPV)	Kruskal-Wallis test	0.03
	wIPV-wIPV-bOPV(-IPV)	VS	wIPV-wIPV-tOPV(-IPV)	Kruskal-Wallis test	>0.9999
			sIPV-bOPV-bOPV(-IPV)	Kruskal-Wallis test	>0.9999
			sIPV-sIPV-bOPV(-IPV)	Kruskal-Wallis test	>0.9999
	wIPV-wIPV-tOPV(-IPV)	VS	sIPV-bOPV-bOPV(-IPV)	Kruskal-Wallis test	>0.9999
			sIPV-sIPV-bOPV(-IPV)	Kruskal-Wallis test	>0.9999
	sIPV-bOPV-bOPV(-IPV)	VS	sIPV-sIPV-bOPV(-IPV)	Kruskal-Wallis test	>0.9999

wIPV：conventional inactivated poliovirus vaccine；sIPV：Sabin strain-based inactivated poliovirus vaccine；tOPV：trivalent oral poliovirus vaccine；bOPV：bivalent oral poliovirus vaccin.

Supplementary Table 7 Multiple comparisons of antibody positive rates against PV2 before booster immunization at 48 months of age

Immunogenicity indicator	Group	VS.	Group	Test method	*P* value
poliovirus type 2	wIPV-bOPV-bOPV	VS.	wIPV-wIPV-bOPV	Fisher exact test	0.321
			wIPV-wIPV-tOPV	Fisher exact test	0.04
			sIPV-bOPV-bOPV	Fisher exact test	0.598
			sIPV-sIPV-bOPV	Fisher exact test	0.511
			sIPV-sIPV-tOPV	Fisher exact test	0.041
	wIPV-wIPV-bOPV	VS.	wIPV-wIPV-tOPV	Fisher exact test	0.401
			sIPV-bOPV-bOPV	Fisher exact test	0.116
			sIPV-sIPV-bOPV	Fisher exact test	0.033
			sIPV-sIPV-tOPV	Fisher exact test	0.403
	wIPV-wIPV-tOPV	VS.	sIPV-bOPV-bOPV	Fisher exact test	0.023
			sIPV-sIPV-bOPV	Fisher exact test	0.001
			sIPV-sIPV-tOPV	Fisher exact test	1
	sIPV-bOPV-bOPV	VS.	sIPV-sIPV-bOPV	Fisher exact test	1
			sIPV-sIPV-tOPV	Fisher exact test	0.023
	sIPV-sIPV-bOPV	VS.	sIPV-sIPV-tOPV	Fisher exact test	0.001

wIPV：conventional inactivated poliovirus vaccine；sIPV：Sabin strain-based inactivated poliovirus vaccine；tOPV：trivalent oral poliovirus vaccine；bOPV：bivalent oral poliovirus vaccin.

Supplementary Table 8 Multiple comparisons of GMT for neutralizing antibody against PV1, PV2, and PV3 before boost immunization at 48 months of age

Immunogenicity indicator	Group	VS.	Group	Test method	*P* value
poliovirus type 1	wIPV-bOPV-bOPV	VS.	wIPV-wIPV-bOPV	Kruskal-Wallis test	>0.9999
			wIPV-wIPV-tOPV	Kruskal-Wallis test	0.0448
			sIPV-bOPV-bOPV	Kruskal-Wallis test	0.0835
			sIPV-sIPV-bOPV	Kruskal-Wallis test	0.1349
			sIPV-sIPV-tOPV	Kruskal-Wallis test	>0.9999
	wIPV-wIPV-bOPV	VS.	wIPV-wIPV-tOPV	Kruskal-Wallis test	0.0076
			sIPV-bOPV-bOPV	Kruskal-Wallis test	0.1115
			sIPV-sIPV-bOPV	Kruskal-Wallis test	0.1797
			sIPV-sIPV-tOPV	Kruskal-Wallis test	>0.9999
	wIPV-wIPV-tOPV	VS.	sIPV-bOPV-bOPV	Kruskal-Wallis test	<0.0001
			sIPV-sIPV-bOPV	Kruskal-Wallis test	<0.0001
			sIPV-sIPV-tOPV	Kruskal-Wallis test	0.0002
	sIPV-bOPV-bOPV	VS.	sIPV-sIPV-bOPV	Kruskal-Wallis test	>0.9999
			sIPV-sIPV-tOPV	Kruskal-Wallis test	0.1833
	sIPV-sIPV-bOPV	VS.	sIPV-sIPV-tOPV	Kruskal-Wallis test	0.3111
poliovirus type 2	wIPV-bOPV-bOPV	VS.	wIPV-wIPV-bOPV	Kruskal-Wallis test	>0.9999
			wIPV-wIPV-tOPV	Kruskal-Wallis test	<0.0001
			sIPV-bOPV-bOPV	Kruskal-Wallis test	>0.9999
			sIPV-sIPV-bOPV	Kruskal-Wallis test	>0.9999
			sIPV-sIPV-tOPV	Kruskal-Wallis test	<0.0001
	wIPV-wIPV-bOPV	VS.	wIPV-wIPV-tOPV	Kruskal-Wallis test	<0.0001
			sIPV-bOPV-bOPV	Kruskal-Wallis test	>0.9999
			sIPV-sIPV-bOPV	Kruskal-Wallis test	>0.9999

			sIPV-sIPV-tOPV	Kruskal-Wallis test	<0.0001
	wIPV-wIPV-tOPV	VS.	sIPV-bOPV-bOPV	Kruskal-Wallis test	<0.0001
			sIPV-sIPV-bOPV	Kruskal-Wallis test	<0.0001
			sIPV-sIPV-tOPV	Kruskal-Wallis test	>0.9999
	sIPV-bOPV-bOPV	VS.	sIPV-sIPV-bOPV	Kruskal-Wallis test	>0.9999
			sIPV-sIPV-tOPV	Kruskal-Wallis test	<0.0001
	sIPV-sIPV-bOPV	VS.	sIPV-sIPV-tOPV	Kruskal-Wallis test	<0.0001
poliovirus type 3	wIPV-bOPV-bOPV	VS.	wIPV-wIPV-bOPV	Kruskal-Wallis test	0.0718
			wIPV-wIPV-tOPV	Kruskal-Wallis test	>0.9999
			sIPV-bOPV-bOPV	Kruskal-Wallis test	>0.9999
			sIPV-sIPV-bOPV	Kruskal-Wallis test	0.1907
			sIPV-sIPV-tOPV	Kruskal-Wallis test	>0.9999
	wIPV-wIPV-bOPV	VS.	wIPV-wIPV-tOPV	Kruskal-Wallis test	0.0011
			sIPV-bOPV-bOPV	Kruskal-Wallis test	>0.9999
			sIPV-sIPV-bOPV	Kruskal-Wallis test	>0.9999
			sIPV-sIPV-tOPV	Kruskal-Wallis test	0.0680
	wIPV-wIPV-tOPV	VS.	sIPV-bOPV-bOPV	Kruskal-Wallis test	>0.9999
			sIPV-sIPV-bOPV	Kruskal-Wallis test	0.0051
			sIPV-sIPV-tOPV	Kruskal-Wallis test	>0.9999
	sIPV-bOPV-bOPV	VS.	sIPV-sIPV-bOPV	Kruskal-Wallis test	>0.9999
			sIPV-sIPV-tOPV	Kruskal-Wallis test	>0.9999
	sIPV-sIPV-bOPV	VS.	sIPV-sIPV-tOPV	Kruskal-Wallis test	0.1536

wIPV：conventional inactivated poliovirus vaccine；sIPV：Sabin strain-based inactivated poliovirus vaccine；tOPV：trivalent oral poliovirus vaccine；bOPV：bivalent oral poliovirus vaccin.

Supplementary Table 9 Multiple comparisons of GMT for neutralizing antibody against PV2 at 28 days after boost immunization at 48 months of age

Immunogenicity indicator	Group	VS.	Group	Test method	*P* value
poliovirus type 2	wIPV-bOPV-bOPV(-bOPV)	VS.	wIPV-wIPV-bOPV(-bOPV)	Kruskal-Wallis test	>0.9999
			wIPV-wIPV-tOPV(-bOPV)	Kruskal-Wallis test	<0.0001
			sIPV-bOPV-bOPV(-bOPV)	Kruskal-Wallis test	>0.9999
			sIPV-sIPV-bOPV(-bOPV)	Kruskal-Wallis test	>0.9999
			sIPV-sIPV-tOPV(-bOPV)	Kruskal-Wallis test	<0.0001
	wIPV-wIPV-bOPV(-bOPV)	VS.	wIPV-wIPV-tOPV(-bOPV)	Kruskal-Wallis test	<0.0001
			sIPV-bOPV-bOPV(-bOPV)	Kruskal-Wallis test	>0.9999
			sIPV-sIPV-bOPV(-bOPV)	Kruskal-Wallis test	>0.9999
			sIPV-sIPV-tOPV(-bOPV)	Kruskal-Wallis test	<0.0001
	wIPV-wIPV-tOPV(-bOPV)	VS.	sIPV-bOPV-bOPV(-bOPV)	Kruskal-Wallis test	<0.0001
			sIPV-sIPV-bOPV(-bOPV)	Kruskal-Wallis test	<0.0001
			sIPV-sIPV-tOPV(-bOPV)	Kruskal-Wallis test	>0.9999
	sIPV-bOPV-bOPV(-bOPV)	VS.	sIPV-sIPV-bOPV(-bOPV)	Kruskal-Wallis test	>0.9999
			sIPV-sIPV-tOPV(-bOPV)	Kruskal-Wallis test	<0.0001
	sIPV-sIPV-bOPV(-bOPV)	VS.	sIPV-sIPV-tOPV(-bOPV)	Kruskal-Wallis test	<0.0001

wIPV：conventional inactivated poliovirus vaccine；sIPV：Sabin strain-based inactivated poliovirus vaccine；tOPV：trivalent oral poliovirus vaccine；bOPV：bivalent oral poliovirus vaccin.
